# Response: Commentary: An *In Silico–In Vitro* Pipeline Identifying an HLA-A*02:01+ KRAS G12V+ Spliced Epitope Candidate for a Broad Tumor-Immune Response in Cancer Patients

**DOI:** 10.3389/fimmu.2021.679836

**Published:** 2021-07-13

**Authors:** Michele Mishto, Guillermo Rodriguez-Hernandez, Jacques Neefjes, Henning Urlaub, Juliane Liepe

**Affiliations:** ^1^ Centre for Inflammation Biology and Cancer Immunology (CIBCI) & Peter Gorer Department of Immunobiology, King’s College London, London, United Kingdom; ^2^ Francis Crick Institute, London, United Kingdom; ^3^ Department of Cell and Chemical Biology, Oncode Institute, Leiden University Medical Center (LUMC), Leiden, Netherlands; ^4^ Max-Planck-Institute for Biophysical Chemistry, Göttingen, Germany; ^5^ Bioanalytics, Institute of Clinical Chemistry, University Medical Center, Göttingen, Germany

**Keywords:** proteasome, peptide splicing, antigen presentation, tumor immunology, mass spectrometry

## Introduction

Proteasomes are the central cytosolic and nuclear proteases in eukaryotic cells. They degrade a broad range of proteins, mainly cytoplasmic, though they are also active in other cell compartments (1). The peptides produced during protein degradation can be further processed by cytosolic aminopeptidases to preserve amino acid availability for new protein synthesis. Not all proteasomal products are destinated for full degradation. For example, p105 is cleaved by proteasomes, thereby generating a component of the transcription factor NF-κB. Similarly, osteopontin seems to be processed by these proteases, which produce peptides that promote cell migration (2–4). However, the best studied peptide products produced by proteasomes are peptides destined for binding HLA-I complexes (*i.e*. the HLA-I immunopeptidome) for presentation at the cell surface to CD8^+^ T cells (5). HLA-I immunopeptidomes have widely been investigated in the context of cancer, radiotherapy, infection, autoimmunity and other conditions. These peptides can be used for detection of specific T cell immune responses and for vaccination purposes against infections and cancer.

**Figure 1:**
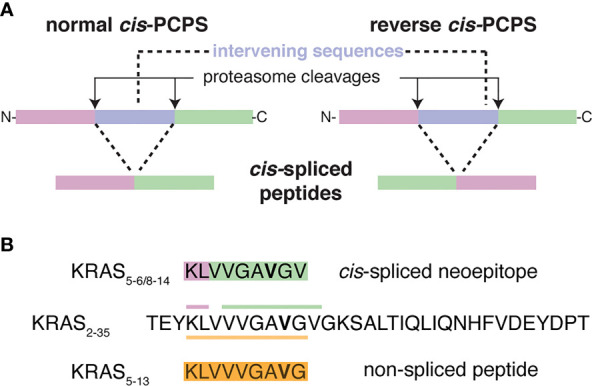
Proteasome-catalyzed peptide splicing and the investigated KRAS G12V *cis*-spliced and non-spliced peptides. **(A)** Proteasome-generated *cis*-spliced peptides can be formed by *cis-*PCPS, when the two splice-reactants, *i.e*. the non-contiguous peptide fragments ligated by proteasomes, derive from the same polypeptide molecule; the ligation can occur in normal order, *i.e.* following the orientation from N- to C-terminus of the parental protein (normal *cis*-PCPS), or in the reverse order (reverse *cis*-PCPS). **(B)** The KRAS_5-6/8-14_ G12V [KL][VVGA**V**GV] *cis*-spliced peptide and the KRAS_5-13_ G12V [KLVVVGA**V**G] non-spliced peptide, which are investigated in this study and derived from the KRAS_2-35_ G12V synthetic substrate polypeptide. The G12V mutation is marked in bold.

It is long established that proteases such as trypsin can both cleave peptide bonds but also ligate (repair) these peptide bonds (6, 7). The proteasome is no exception and can produce so-called spliced peptides through proteasome-catalyzed peptide splicing (PCPS). This latter process can occur by combining non-contiguous peptide fragments of either the same molecule – *i.e*. *cis*-PCPS – or of two distinct proteins - *i.e. trans*-PCPS (**Figure 1A**). This results in peptide sequences that are not defined by the genetic code. *Cis*-spliced peptide identification is challenging: depending on the method of identification used, their estimated frequency in HLA-I immunopeptidomes ranged from 1% to 34% (8). PCPS is not a random process and seems to be ruled by sequence motif preferences and driving forces, which may differ from peptide hydrolysis, although the exact rules are largely unknown (7, 9–11). *Cis*-spliced peptides seem to be routinely produced and presented by various cells, depicting peptide splicing as a standard activity of proteasomes (7, 9, 12–17). On average, they are produced and presented by HLA-I complexes in smaller amount than non-spliced peptides although exceptions have been described (10, 12, 15, 17). *Cis*-spliced peptides can target *in vivo* CD8^+^ T cell response against bacterial antigens otherwise neglected in a mouse model of *Listeria monocytogenes* infection (18). They can activate *ex vivo* CD8^+^ T cells specific to *Listeria monocytogenes* or HIV through cross-recognition (19, 20). *Cis*-spliced epitopes derived from melanoma-associated antigens are recognized by CD8^+^ T cells in peripheral blood of melanoma patients (13, 17). A melanoma patient with metastasis was cured through adoptive T cell therapy using an autologous tumor-infiltrating lymphocyte (TIL) clone, which was proved, in a later study, to be specific to a *cis*-spliced epitope rather than any non-spliced peptides derived from the melanoma-associated antigen tyrosinase (21, 22). Despite their theoretically vast sequence variability, *cis*-spliced peptides might not play a destabilizing role in central and peripheral tolerance and in pruning T cell repertoire (23), although they may be involved in autoreactive CD8^+^ T cell response in autoimmune diseases such as Type 1 Diabetes (T1D) (24, 25).

Since the discovery of the first two proteasome-generated *cis*-spliced epitopes in 2004, mass spectrometry (MS) represented a cornerstone of the investigation of PCPS (26, 27). Although in the first years the identification of *cis*-spliced peptides was dependent on the availability of CD8^+^ T cell clones (often TILs), in 2010 the development of the first bioinformatics strategy, named SpliceMet (28), for the identification of spliced peptides *in vitro* digestions of synthetic polypeptides heralded a new era. Since then, thousands of *cis*-spliced peptides have been identified through the combination of MS and bioinformatics in this type of assays (10, 11, 29–31).

Since their discovery, *cis*-spliced epitopes have been studied in relation to tumor immunology, as alternative sources for attacking cancer cells through adoptive T cell therapies. Particularly attractive was the idea of exploiting the theoretically large variability of *cis*-spliced peptide sequences to target those recurrent driver mutations that are poorly presented *via* non-spliced peptides onto predominant HLA-I variants (5). For instance, KRAS is frequently mutated, especially in colorectal cancer and pancreatic adenocarcinoma, wherein 33-61% of the patients carry a KRAS mutation (32). The mutations often occur in the KRAS G12 and G13 residues, which impair the KRAS GTPase activity and render the mutants persistently in the GTP-bound active form, thereby driving carcinogenesis (33). Since KRAS G12/13 mutations are highly frequent, often clonal, and driver mutations in tumors, they are ideal targets for immunotherapies, as underlined by the successful pioneering studies of Rosenberg and Yang laboratories (34, 35). In 2019, we investigated the targeting of these mutations in HLA-A*02:01^+^ patients, since this HLA-I allele is present in approximately 50% of the Caucasian population. Through the development of an *in silico/in vitro* pipeline, we confirmed that no non-spliced epitope candidates carrying KRAS G12/13 mutations could be presented by HLA-A*02:01 with predicted IC50 ≤ 100 nM. By contrast, many *cis*-spliced epitope candidates carrying these mutations might be produced by proteasomes (11). We focused on the KRAS G12V mutation and identified, through targeted MS, the KRAS_5-6/8-14_ G12V [KL][VVGA**V**GV] *cis*-spliced peptide in 20 h *in vitro* digestion of the synthetic KRAS_2-35_ G12V polypeptide with purified proteasomes. Through the application of various *in vitro* techniques, we and others demonstrated that this tumor-specific *cis*-spliced neoepitope candidate can efficiently (i) be produced by proteasomes in *in vitro* kinetics experiments, (ii) be transported by Transporters Associated with antigen Processing (TAP) heterodimers into the endoplasmic reticulum (ER) lumen, and (iii) bind HLA-A*02:01 complex (11).

The central outcome in this study was, of course, the production of the KRAS_5-6/8-14_ G12V *cis*-spliced peptide by proteasomes, which was proved by targeted MS (11).

In his matters arising, Dr. Beer disputed such identification and he suggested that “the *in vitro* proteasome digestion products did not include the spliced peptide and therefore further analysis relied on incorrect identification” (36). Notably, he claimed that the KRAS_5-6/8-14_ G12V *cis*-spliced peptide was incorrectly identified, and should instead be the non-spliced peptide KRAS_5-13_ G12V [KLVVVGA**V**G] (see **Figure 1B**), and that our MS measurements could be the result of a specific kind of contamination. To support his hypothesis, he reanalyzed some of our MS RAW files available on PRIDE repository, he measured the synthetic KRAS_5-13_ G12V non-spliced peptide on a different MS instrument and reconstructed a timeline of our original study, which strongly differed from what we presented in Mishto et al. (11).

We dispute Dr. Beer’s suggestions and address them through an evaluation of his analysis. In addition, we present the true timeline of our original study, and comprehensive details of which samples were measured when, and through which MS instrument. We also provide new *in vitro* experiments, MS measurements and analyses. These data confirm that the KRAS_5-6/8-14_ G12V *cis*-spliced peptide is indeed produced *in vitro* by proteasomes, as we previously concluded, and resolves the misunderstandings of our data expressed by Dr. Beer.

## Materials and Methods

### Peptide Synthesis and Proteasome Purification

All peptides were synthesized using Fmoc solid phase chemistry. 20S proteasomes were purified from peripheral blood as described elsewhere (11). Proteasome concentration was measured by Bradford staining and verified by Coomassie staining of an SDS-Page gel, as shown elsewhere. The purity of the preparation using this protocol has been previously shown (31).

### *In Vitro* Digestions and MS Measurements

In our original study (11), the synthetic KRAS_2-35_ G12V polypeptide (sequence: TEYKLVVVGA**V**GVGKSALTIQLIQN HFVDEYDPT; 40 µM final concentration) was digested by 3 µg 20S proteasomes (purified from peripheral blood) in 100 µl TEAD (20 mM Tris/KOH-pH 7.2, 1 mM EDTA, 1 mM DTT, 0.02% Sodium azide) buffer for different time points (0-4 h and 20 h) at 37°C. We performed three independent experiments, each of them measured either 3 times (for the 0-4 h kinetics) or 2 times (for 20h digestions) by MS. In the present study, the synthetic KRAS_2-35_ G12V polypeptide (40 µM final concentration) was digested by 1.6 µg 20S proteasomes (purified from human erythrocytes) for different time points (0-4 h) at 37°C in 40 µl HMDA buffer (20 mM Hepes/KOH-pH 7.2, 5 mM MgCl2, 1 mM DTT, 0.02% sodium azide; samples “MF”) or TMDA (20 mM Tris/KOH-pH 7.2, 5 mM MgCl2, 1 mM DTT, 0.02% Sodium azide; samples “MG”), as previously described (37). We generated one biological sample per condition, which was measured once per mass spectrometer.

In our original study (11), the identification of KRAS_5-6/8-14_ G12V *cis*-spliced peptide was carried out by targeted mass spectrometry (MS) using a mass to charge ratio (m/z) inclusion list. 20 h *in vitro* digestions with 20S proteasomes were measured by Fusion Lumos Mass Spectrometer (Thermo Fisher Scientific). By contrast, the quantification of the target peptides was carried out by measuring *in vitro* proteasome-mediated digestion kinetics (0-4 h) through a Q Exactive Orbitrap mass spectrometer. Prior to measurement, the samples were diluted with the loading buffer (2% acetonitrile, 0.05% Trifluoroacetic acid) containing human insulin (Sigma-Aldrich), as explained elsewhere (11).

In the present study, the identification of KRAS_5-6/8-14_ G12V *cis*-spliced peptide and the KRAS_5-13_ G12V non-spliced peptide was carried out by standard MS. In particular, *in vitro* proteasome-mediated digestion kinetics (0 and 4 h) were measured by injecting 8 µl sample in the Exploris 480 Orbitrap coupled to an Ultimate 3000 RSLC nano pump (both from Thermo Fisher Scientific). Samples were loaded and separated by a nanoflow HPLC (RSLC Ultimate 3000) on an Easy-spray C18 nano column (30 cm length, 75 µm internal diameter; Dr. Maisch) coupled on-line to a nano-electrospray ionization Orbitrap Exploris 480 mass spectrometer (Thermo Fisher Scientific). Peptides were eluted with a linear gradient of 5%–55% buffer B (80% ACN, 0.1% formic acid) over 88 min at 50°C at a flow rate of 300 nl/min. The instrument was programmed within Xcalibur 3.1.66.10 to acquire MS data in a Data Dependent Acquisition mode using Top 20 precursor ions. We acquired one full-scan MS spectrum at a resolution of 120,000 with an automatic gain control (AGC) target value of 1,000,000 ions and a scan range of 350~1600 m/z. The MS/MS fragmentation was conducted using HCD collision energy (30%) with an Orbitrap resolution of 30,000 at 1.4 m/z isolation window with Fixed First Mass set to 110 m/z. The AGC target value was set up at 100,000 with a maximum injection time of 25 ms. For Data Dependent Scans the minimum AGC target value and the Intensity threshold were set to 2,600 to 20,000 accordingly. A dynamic exclusion of 22 s and 1-6 included charged states were defined within this method.

Furthermore, *in vitro* proteasome-mediated digestion kinetics (0 - 4 h) were measured by injecting 5 µl sample in the Q Exactive HF Orbitrap coupled to an Ultimate 3000 RSLC nano pump (both from Thermo Fisher Scientific). Samples were loaded and separated by a nanoflow HPLC (RSLC Ultimate 3000) on an Easy-spray C18 nano column (31cm length, 75 µm internal diameter; Dr. Maisch) coupled on-line to a nano-electrospray ionization Q Exactive HF Orbitrap mass spectrometer (Thermo Fisher Scientific). Peptides were eluted with a linear gradient of 5%–55% buffer B (80% ACN, 0.1% formic acid) over 88 min at 50°C at a flow rate of 3 µl/min. The instrument was programmed within Xcalibur 3.1.66.10 to acquire MS data in a Data Dependent Acquisition mode using Top 30 precursor ions. We acquired one full-scan MS spectrum at a resolution of 60,000 with an automatic gain control (AGC) target value of 1,000,000 ions and a scan range of 200~2,000 m/z. The MS/MS fragmentation was conducted using HCD collision energy (30%) with an Orbitrap resolution of 15,000 at 1.6 m/z isolation window with Fixed First Mass set to 110 m/z. The AGC target value was set up at 100,000 with a maximum injection time of 60 ms. For Data Dependent Scans the minimum AGC target value and the Intensity threshold were set to 2500 to 42000 accordingly. A dynamic exclusion of 30 s and 2-6 included charged states were defined within this method.

Samples were also prepared and measured through Q Exactive Orbitrap mass spectrometer, as described in the original paper (11), to reproduce the measurement conditions - and to be compared to the data - of the original study (11).

In each series of measurement, the digestion samples were measured prior to the synthetic peptides, as we did in the original study (11).

### Spliced and Non-Spliced Peptide Identification and Quantification

Peptides were identified using the Mascot version 2.6.1 (Matrix Science) search engine. Mass spectra were searched against a customized database that includes all theoretically possible spliced and non-spliced peptides (28). M oxidation and N/Q deamidation were set as variable modification. For the peptide identification and quantification in the Q Exactive Orbitrap and in the Q Exactive HF Orbitrap measurements, we set 6 ppm and 20 ppm as mass tolerances for MS and MS2, respectively. For the peptide identification we set 5 ppm and 0.02 Da as mass tolerances for MS and MS2, respectively, in the Exploris 480 and Fusion Lumos Orbitrap measurements.

For the identification, peptide hits were filtered using an ion score cut-off of 20, a q-value cut off of 0.05 and a delta score between two spliced peptide hits of 10% or between a top scoring spliced peptide and a lower scoring non-spliced peptide of 30% (30). Mascot Distiller’s label-free quantification toolbox was used to automatically extract MS ion peak areas of all identified peptides for all 5 time points (0 - 4 h). KRAS_5-6/8-14_ G12V and KRAS_5-13_ G12V peptide generation kinetics were manually quantified by extraction of an ion chromatogram (XIC) corresponding to the peptide monoisotopic peaks, using instrument precursor tolerance and retention time information (from the measured retention time of the synthetic peptide analog) *via* Mascot Distiller, followed by determination of the area under the peak at each time point in the kinetics series.

### Statistical Analysis

Standard proteomics statistics have been applied through the use of the specified MS software.

### Dataset and Software Availability

The MS data have been deposited to the ProteomeXchange Consortium *via* the PRIDE (38) partner repository with the dataset identifier PXD015580, associated to Mishto et al. (11), and PDX024528, associated to the present manuscript.

Beer’s MS data and methods have been deposited to the ProteomeXchange Consortium *via* the PRIDE partner repository with the dataset identifier PXD023922.

## Results

We grouped our results to respond to the three issues from Dr. Beer, which comprise his matters arising: the MS2 spectra identified by Mishto and colleagues (11) as KRAS_5-6/8-14_ G12V *cis*-spliced peptide appeared to have been misidentified (i), and they belonged to the KRAS_5-13_ G12V non-spliced peptide (ii); (iii) the KRAS_5-6/8-14_ G12V *cis*-spliced peptide, which was found in follow up analyses by Mishto and colleagues (11), was suspected to be a specific kind of contamination.

### The Spliced Neoepitope KRAS_5-6/8-14_ G12V Is Produced by Proteasomes and Its Identification Is Favored by the *In Silico/In Vitro* Pipeline We Originally Proposed

**Figure 2:**
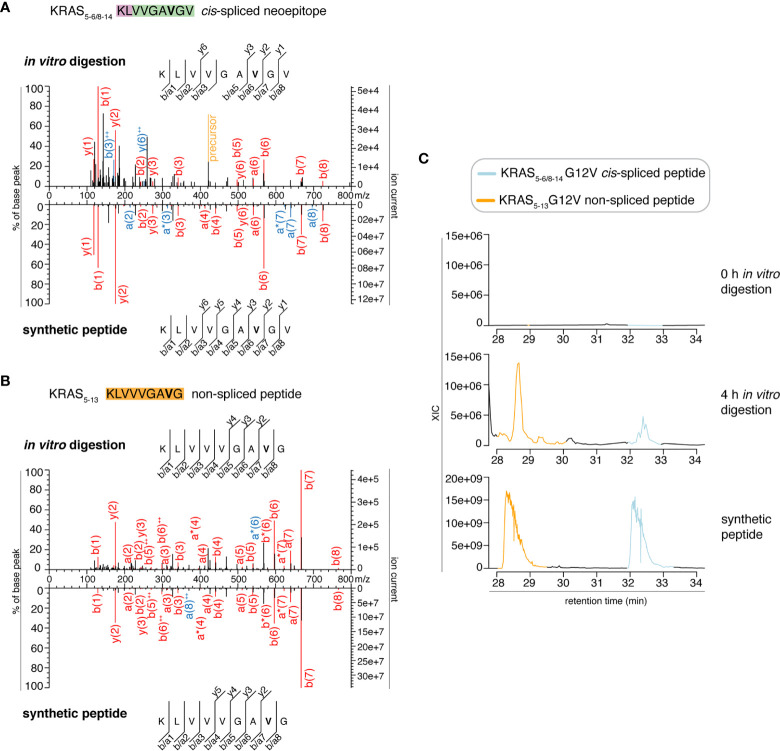
MS2 spectra of the KRAS_5-6/8-14_ G12V *cis*-spliced and KRAS_5-13_ G12V non-spliced peptides by standard MS through Exploris 480 Orbitrap. **(A, B)** MS2 spectra of the KRAS_5-6/8-14_ G12V [KL][VVGAVGV] *cis*-spliced peptide **(A)** and KRAS_5-13_ G12V non-spliced peptide [KLVVVGAVG], which were identified in the 4 h *in vitro* digestions of the synthetic polypeptide KRAS_2-35_ G12V with purified proteasomes, in comparison with the cognate synthetic peptides. The peptide sequence is shown with the corresponding b-, a- and y-ions identified. The G12V mutation is depicted in bold. In the spectra, assigned peaks for b-, a- and y-ions are reported in color. Ion neutral loss of ammonia and double charged ions are symbolized by * and ^++^, respectively. Red marked peaks are assigned in MS2 spectra of both *in vitro* digestions and synthetic peptides, whereas blue marked peaks are assigned only in one of the two spectra. We marked the doubly charged peptide precursor in yellow. **(C)** Extracted ion chromatograms for the target peptides in the 0 h and 4 h in *vitro* digestion and the synthetic counterpart are plotted and indicate matching RTs and absence of a biologically significant peak in the 0 h digestion. MS ion chromatograms correspond to the m/z = 421.275 – 421.875. In **(A–C)** samples correspond to MF series carried out in HMDA buffer and have been measured through standard MS by Orbitrap Exploris 480 mass spectrometer.

**Figure 3:**
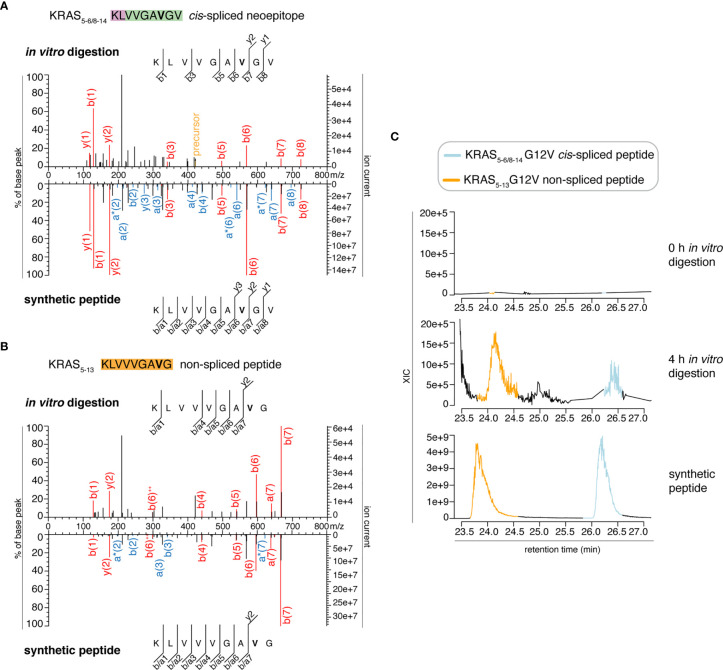
Identification and quantification of KRAS_5-6/8-14_ G12V *cis*-spliced and KRAS_5-13_ G12V non-spliced peptides in *in vitro* digestion kinetics by standard MS through Q Exactive HF Orbitrap. **(A, B)** MS2 spectra of the KRAS_5-6/8-14_ G12V [KL][VVGAVGV] *cis*-spliced peptide **(A)** and KRAS_5-13_ G12V non-spliced peptide [KLVVVGA**V**G], which were identified in the 4 h *in vitro* digestions of the synthetic polypeptide KRAS_2-35_ G12V with purified proteasomes, in comparison with the cognate synthetic peptides. The peptide sequence is shown with the corresponding b-, a- and y-ions identified. The G12V mutation is depicted in bold. In the spectra, assigned peaks for b-, a- and y-ions are reported in color. Ion neutral loss of ammonia and double charged ions are symbolized by * and ^++^, respectively. Red marked peaks are assigned in MS2 spectra of both *in vitro* digestions and synthetic peptides, whereas blue marked peaks are assigned only in one of the two spectra. We marked the doubly charged peptide precursor in yellow. **(C)** Extracted ion chromatograms for the target peptides in the 0 h and 4 h *in vitro* digestion and the synthetic counterpart are plotted and indicate matching RTs and absence of a biologically significant peak in the 0 h digestion. MS ion chromatograms correspond to the m/z = 421.275 – 421.875. In **(A–C)** samples correspond to MF series carried out in HMDA buffer and have been measured through standard MS by Q Exactive HF Orbitrap mass spectrometer.

In our original paper (11), we developed an *in silico* pipeline able to predict translationally relevant neoantigens (section 1) and to suggest non-spliced and *cis*-spliced neoepitope candidates carrying target mutations within those neoantigens [section 2; for details, see Figures 1 and 2 in (11)]. Our pipeline had a third section, consisting in a validation of neoepitope candidates by *in vitro* assays recapitulating some of the main steps of the HLA-I antigen processing and presentation (APP) pathway. The first step of the third section was to investigate the production of the neoepitope candidate by purified proteasomes. Section 2 of our *in silico* pipeline provided the sequence of the peptide candidates, which we used for a targeted MS carried out with the most sensitive Orbitrap equipment that we had at the time, *i.e*. Fusion Lumos Mass Spectrometer. Through this strategy we identified the KRAS_5-6/8-14_ G12V *cis*-spliced peptide after 20 h digestion of the synthetic polypeptide KRAS_2-35_. We predicted that (almost) all substrate molecules were processed by proteasomes after 20 h, and thus we would have the highest likelihood to obtain an excellent MS2 spectrum. This was the case, and the comparison of the MS2 spectra of the KRAS_5-6/8-14_ G12V *cis*-spliced peptide and its cognate synthetic peptide confirmed the production of the peptide by proteasomes [see Figure 3 in (11)]. All cognate files are available on PRIDE repository (PXD015580).

After identifying the target peptide, we measured its generation over time through MS measurement of *in vitro* digestion kinetics (0 - 4 h) of the synthetic KRAS_2-35_ G12V polypeptide with purified proteasomes. The conditions were set to still have unprocessed substrate molecules after 4 h, which is a standard procedure in this type of experiment. In our original paper (11), we published 45 MS Raw files, which recapitulated three biological replicates, 5 time points each (0, 1, 2, 3, 4 h) with three technical replicates each, as well as the cognate result files, as obtained by Mascot analysis. All cognate files are available on PRIDE repository (PXD015580). In contrast to the 20 h digestion samples, these samples were measured by a Q Exactive Orbitrap mass spectrometer without using a m/z inclusion list. We used this latter mass spectrometer, despite its lower mass resolution than the Fusion Lumos Orbitrap, for practical reasons (*e.g*. shorter user queue and hence more measurement and optimization opportunities). We did not apply a m/z inclusion list because we aimed to quantify the target peptide as well as all other peptide products, thereby shedding light on the PCPS dynamics compared to peptide hydrolysis [see Figure 5A in (11) for details of the outcomes]. Therefore, we expected to not have an informative MS2 spectrum for the KRAS_5-6/8-14_ G12V *cis*-spliced peptide in this set of MS measurements, and the identification of the *cis*-spliced peptide’s peak for quantification was done through m/z and retention time (RT) comparison with the cognate synthetic peptide.

In summary, the targeted MS measurement (using a m/z inclusion list) of the 20 h digestion samples with Fusion Lumos Orbitrap aimed toward peptide identification whereas the standard MS measurement with Q Exactive Orbitrap of the kinetic digestions aimed towards quantification of peptide production (including neoepitope candidates). These details were clearly explained in our original paper (11).

Nonetheless, Dr. Beer performed an analysis of our *in vitro* digestion kinetics (0 - 4 h), wherein he failed to identify the KRAS_5-6/8-14_ G12V *cis*-spliced peptide; and he claimed that this proved that the peptide was not produced by proteasomes at all. Dr. Beer (36) shows: in Figure 1A (36), the MS2 spectrum of the KRAS_5-6/8-14_ G12V *cis*-spliced synthetic peptide that we originally obtained through Fusion Lumos Orbitrap targeted measurement; in Figures 1B–D (36), three MS2 spectra (MS scans 11558, 11683, 11389) of peptide products, which we originally obtained through Q Exactive Orbitrap standard measurement of the kinetic digestions (4 h); in Figure 1E (36), the MS2 spectrum of the KRAS_5-13_ G12V non-spliced peptide that Dr. Beer obtained through Q Exactive Plus’s standard measurement of the cognate synthetic peptide.

Dr. Beer subsequently stated that we identified the KRAS_5-6/8-14_ G12V *cis*-spliced peptide in 34 out of 45 MS runs of the *in vitro* digestion kinetics (0-4 h) measured through Q Exactive Orbitrap mass spectrometer (36). His claim was based on the Mascot result files F011920.csv, F011921.csv, and F011929.csv, which we published in our PRIDE dataset (PXD015580).

Dr. Beer’s claim that we identified the KRAS_5-6/8-14_ G12V *cis*-spliced peptide in these MS2 spectra, which are shown in Dr. Beer’s Figures 1B-D (36), is simply incorrect. Indeed, as explained above and stated in our original paper (11), we identified the KRAS_5-6/8-14_ G12V *cis*-spliced peptide in the 20 h digestion samples through Fusion Lumos Orbitrap targeted MS. Since no further information is provided by Dr. Beer, we can only speculate that Dr. Beer misunderstood the nature of the Mascot result files that we published on PRIDE repository and were obtained through Q Exactive Orbitrap mass spectrometer. Those Mascot result files are the original search result files created by Mascot, which have been published unmodified on the PRIDE repository, according to the repository’s guidelines. These search result files were therefore unfiltered and contained up to ten peptide sequence suggestion per MS2 scan. In our original study (11), we applied our stringent identification method to filter those files and none of the recorded MS2 scans were then identified as the KRAS_5-6/8-14_ G12V *cis*-spliced peptide. Our peptide candidate analysis strategy was explained in detail in the Materials and Methods as follows “peptide hits were filtered using an ion score cut-off of 20, a q-value cut off of 0.05 and a delta score between two spliced peptide hits or between a top scoring spliced peptide and a lower scoring non-spliced peptide of 30%” (11). None of the three MS2 scans, shown by Dr. Beer in his Figures 1B-D (36), were assigned by us to a peptide sequence, because all hits failed to pass this identification threshold (**Table S1**). Furthermore, in all three MS2 scans, the KRAS_5-6/8-14_ G12V *cis*-spliced peptide was not the best hit (*i.e*. the only peptide with rank 1), which makes it difficult to understand Dr. Beer’s claims. He asserted that these “three scans faithfully represent all the spectra identified by the authors as KLVVGAVGV in the 34 runs”. Since we did not identify the KRAS_5-6/8-14_ G12V *cis*-spliced peptide from those scans in our original study (11), the basis of Beer’s first claim is unsubstantiated.

Furthermore, we would like to mention that, in proteomics, it is good practice to compare MS2 of peptides identified by using the same mass spectrometer and methods settings. We followed this principle in our original paper [see Figure 3 in (11)] and in the MS2 spectra shown in this rebuttal article. By contrast, Dr. Beer combined MS2 spectra obtained through various MS methods implemented on Fusion Lumos Orbitrap and Q Exactive Orbitrap (our measurements) and through Q Exactive Plus Orbitrap (his measurement) in his Figure 1 (36), which may be misleading.

In the present study, we tried to identify the KRAS_5-6/8-14_ G12V *cis*-spliced peptide in proteasomes-catalyzed kinetic digestions without using the targeted MS approach but by exploiting the higher sensitivity of latest generation mass spectrometers. To this end, we performed *in vitro* digestion kinetics (0 - 4 h) of the synthetic KRAS_2-35_ G12V polypeptide with purified proteasomes. In this set of experiments, we used two different buffers (“MF” series used HMDA buffer and “MG” series used TMDA buffer) than those originally used, and a different proteasome source (see Material & Method for details), to verify whether the KRAS_5-6/8-14_ G12V *cis*-spliced peptide could also be produced by proteasomes in different *in vitro* conditions. The samples were then measured through Exploris 480 and Q Exactive HF Orbitraps, without a targeted m/z. This latter approach may help us to understand whether our *in silico* pipeline for neoepitope candidate identification may become redundant when researchers have access to more accurate and sensitive mass spectrometers. Through Exploris 480 Orbitrap measurements of “MF” series, we did not identify MS2 spectra for either the KRAS_5-6/8-14_ G12V *cis*-spliced or the KRAS_5-13_ G12V non-spliced peptides in t = 0 h, whereas we identified MS2 spectra for both peptides in t = 4 h (**Figures 2A, B**). In the 4 h digestion experiments, the MS2 spectra of both peptides had MS scores that passed our identification threshold (**Table S2**). Their assignment was also confirmed through comparison of the RT of the *in vitro* digestions and the synthetic peptides in the mass-specific ion chromatograms (**Figure 2C**). Significantly, the two peptides had distinct elution times in this MS set up, which allows us to clearly assign MS2 spectra in combination with elution times, making the hypothesis of mistaking one peptide for the other extremely unlikely, contrary to Dr. Beer’s suggestion (see below).

We then extended the measurements to the entire 0 – 4 h *in vitro* digestion kinetics of both “MF” and “MG” series by using a Q Exactive HF Orbitrap. We identified MS2 spectra of the KRAS_5-6/8-14_ G12V *cis*-spliced after 2, 3 and 4 h digestion of the synthetic KRAS_2-35_ G12V polypeptide with purified proteasomes in the “MF” series and after 3 h in the “MG” series (**Figure 3A** and **Table S2**). MS2 spectra of the KRAS_5-13_ G12V non-spliced peptide were identified in the “MF” series (**Figure 3B** and **Table S2**). In both series, both peptides were not identified at t = 0 or in the absence of proteasomes. Even in these latter measurements, the peptide identification was confirmed by RT matching with a synthetic peptide; the two peptides showed a distinct elution profile, which excludes the possibility of mistaking one peptide for the other (**Figure 3C** and **Figure S1**).

To note, although the present experiments and those previously published (11) have been carried out with different conditions, the excellent ion coverage of the target *cis*-spliced neoepitope measured with targeted MS (Figure 3 in (11) and section below) indicates that this approach, and thus our *in silico* pipeline (11), is sufficiently robust for peptide identification, and withstands measurements through more sensitive mass spectrometers.

Collectively, we show that: (a) Dr. Beer’s first claim was incorrect, (b) the KRAS_5-6/8-14_ G12V *cis*-spliced peptide was produced by proteasomes in the kinetic digestions, and (c) our *in silico/in vitro* pipeline is proper for the identification of neoepitope candidates, which was the main message of our original study (11).

### The KRAS_5-13_ G12V Non-Spliced Peptide Is Produced by Proteasomes Although It Is Not an Alternative Identification of the *Cis*-Spliced Neoepitope KRAS_5-6/8-14_ G12V

In his matters arising, Dr. Beer (36) claimed that some of the MS2 spectra derived from 4 h digestions - and measured through Q Exactive Orbitrap - were used by us for the identification of the KRAS_5-6/8-14_ G12V *cis*-spliced peptide [KL][VVGA**V**GV]. He also claimed that, in reality, those MS2 spectra belonged to the KRAS_5-13_ G12V non-spliced peptide [KLVVVGA**V**G], and thus we misassigned their sequence. As discussed above, in our original study (11), we identified the KRAS_5-6/8-14_ G12V *cis*-spliced peptide using different samples (*i.e*. 20 h digestions) and a Fusion Lumos Orbitrap rather than a Q Exactive Orbitrap. Dr. Beer stated “this peptide (*i.e*. KRAS_5-13_ G12V non-spliced peptide) has the same mass as the presumed spliced peptide KLVVGAVGV and was among the possible identification of the spectra in question by the Mascot search engine (e.g. in file F011929.csv). However, the authors opted not to choose this identification”. As clarified above, we did not assign any sequence to those MS2 scans chosen by Dr. Beer from the Q Exactive Orbitrap measurements. Even if we had, the KRAS_5-6/8-14_ G12V *cis*-spliced peptide would not have been the assigned sequence since it was not the best hit (**Table S1**).

Nonetheless, it is always possible that we accidentally integrated the incorrect ion peak for the quantification of the KRAS_5-6/8-14_ G12V *cis*-spliced peptide in the *in vitro* digestion kinetics measured by Q Exactive Orbitrap, which we originally published in Figure 5D (11). The integrated ion peaks might have belonged to the KRAS_5-13_ G12V non-spliced peptide, which has the same molecular weight of the KRAS_5-6/8-14_ G12V *cis*-spliced peptide. Of course, this error might have been more likely if the two peptides had the same RT in the MS HPLC chromatogram. Dr. Beer claimed that the KRAS_5-13_ G12V non-spliced peptide could be the actual peptide, in some of the MS scans that he incorrectly interpreted as identified by us as the KRAS_5-6/8-14_ G12V *cis*-spliced peptide. He supported his hypothesis by running the synthetic KRAS_5-13_ G12V non-spliced peptide (but neither the *in vitro* digestions nor the synthetic KRAS_5-6/8-14_ G12V *cis*-spliced peptide) on a different mass spectrometer and chromatography device than the one used by us.

In order to perform a proper comparison, we measured the synthetic peptides of both KRAS_5-6/8-14_ G12V [KL][VVGA**V**GV] *cis*-spliced peptide and KRAS_5-13_ G12V [KLVVVGA**V**G] non-spliced peptide after the measurement of the new *in vitro* digestion kinetics (“MF” and “MG” series) by both Exploris and Q Exactive HF Orbitraps (**Figures 2** and **3**). In both mass spectrometers’ measurements, the two synthetic peptides had a different RT (**Figures 2** and **3**), which nullified the hypothesis of mistaking one peptide for the other, as suggested by Dr. Beer. Furthermore, in the new kinetics, we identified both peptides with MS2 above our original identification threshold (**Figures 2** and **3**), demonstrating that both peptides can be produced by proteasomes. The different MS2 scans, different MS2 fragmentation pattern and different RT are sufficient to refute that we assigned the KRAS_5-6/8-14_ G12V *cis*-spliced peptide sequence to a MS2 spectrum belonging to KRAS_5-13_ non-spliced G12V peptide.

**Figure 4:**
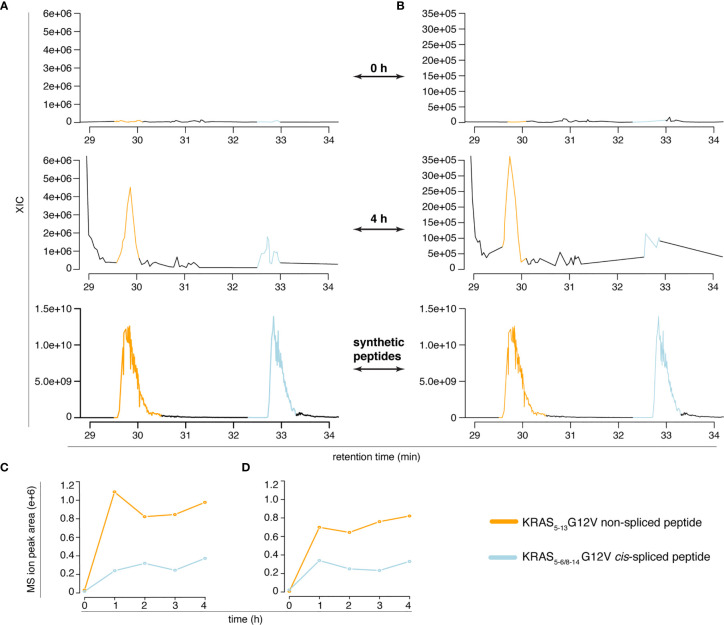
Quantification of KRAS_5-6/8-14_ G12V *cis*-spliced and KRAS_5-13_ G12V non-spliced peptides in *in vitro* digestion kinetics. **(A, B)** MS extracted ion chromatograms of the target peptides in the *in vitro* digestion kinetics of the “MF” **(A)** and “MG” **(B)** series (0 h and 4 h) and of the mix of the two synthetic peptides. **(C, D)** Quantification of the MS ion peak area of the KRAS_5-6/8-14_ G12V *cis*-spliced and KRAS_5-13_ G12V non-spliced peptides in the *in vitro* digestion kinetics of the “MF” **(C)** and “MG” **(D)** series. In **(A–D)** *in vitro* digestion kinetics of the synthetic polypeptide KRAS_2-35_ G12V with purified proteasomes using either HMDA (“MF” series) or TMDA (“MG” series) are shown. Samples have been measured through Q Exactive Orbitrap (one technical replicate each condition). The MS ion chromatograms correspond to the m/z = 421.275 – 421.875.

**Figure 5:**
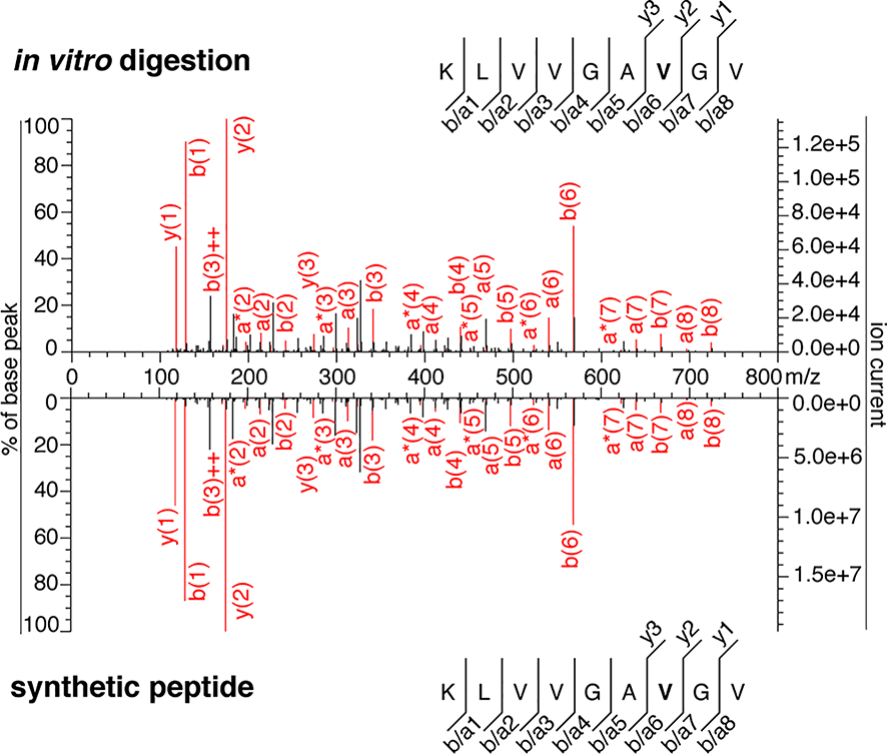
MS2 spectra of the KRAS_5-6/8-14_ G12V *cis*-spliced peptide. MS2 spectrum of the peptide KRAS_5-6/8-14_ G12V [KL][VVGA**V**GV] identified in an *in vitro* 20* h* digestion of the synthetic polypeptide KRAS_2-35_ G12V with proteasomes and of the cognate synthetic peptide. The *in vitro* 20h digestion was performed months earlier than the assays shown in Figures 3–5 of (11). This *in vitro* 20* h* digestion and the cognate synthetic peptide were measured through the Fusion Lumos Orbitrap months earlier than the assays shown in Figures 4 and 5 of (11). The peptide sequence is shown with the corresponding b-, a- and y-ions identified. The G12V mutation is depicted in bold. In the spectra, assigned peaks for b-, a- and y-ions are reported in red. Ion neutral loss of ammonia is symbolized by * and double charges by ++.

However, the two peptides might have had a similar elution profile in the Q Exactive Orbitrap measurements of *in vitro* digestion kinetics that we originally published (11), which might have caused an erroneous quantification of the peptide generation kinetics. To test this option, we measured the new *in vitro* digestion kinetics (“MF” and “MG” series) through the same Q Exactive Orbitrap as used in our original paper (11). Even in this MS set up, the synthetic KRAS_5-6/8-14_ *cis*-spliced and KRAS_5-13_ non-spliced G12V peptides showed a different RT in the ion chromatograms (**Figure 4** and **Figure S2**). This allowed a quantification of their production in the new *in vitro* kinetics (**Figure 4**), and confirmed the correct quantification of the KRAS_5-6/8-14_ *cis*-spliced peptide as presented in our original study [Figure 5D of (11)].

Regarding data sharing, in the PRIDE repository (PXD015580) associated to the original study (11), we provided the MS RAW files of all biological and technical replicates of the *in vitro* digestion kinetics and of the synthetic KRAS_5-6/8-14_ *cis*-spliced G12V peptide measured through Q Exactive Orbitrap. Here, we provided the MS RAW files of the former titration of the KRAS_5-6/8-14_ *cis*-spliced G12V peptide through Q Exactive Orbitrap in the new PRIDE repository dataset associated to this rebuttal article (PDX024528). The readers can compare these files to the files of the *in vitro* kinetics digestions previously published on PRIDE repository (PXD015580).

In summary, in this section, we provide data showing that: (a) Dr. Beer’s second claim is incorrect, (b) both KRAS_5-6/8-14_ G12V *cis*-spliced and KRAS_5-13_ non-spliced G12V peptides can be separated and identified, and were produced by proteasomes in the *in vitro* kinetic digestions in quantified fashion, and (c) our previous quantification of the KRAS_5-6/8-14_ G12V *cis*-spliced peptide in the *in vitro* kinetic digestions was correct (11).

### The KRAS_5-6/8-14_ G12V *Cis*-Spliced Neoepitope Identification Is Not an Outcome of a Specific Kind of Contamination and the Timeline Reconstruction of Our Study by Dr. Beer Is Incorrect

As explained in the previous section, our original *in silico/in vitro* pipeline (11) supported the identification of *cis*-spliced neoepitope candidates. In that study, we used the resulting list of peptide candidates for a targeted MS using Fusion Lumos Orbitrap, whereby we measured the 20 h *in vitro* digestions of the synthetic KRAS_2-35_ G12V polypeptide with purified proteasomes. This step led to the identification of the target KRAS_5-6/8-14_ G12V *cis*-spliced peptide. Afterwards, we measured the 0-4 h *in vitro* digestion kinetics of the synthetic KRAS_2-35_ G12V polypeptide with purified proteasomes and quantified the production of the KRAS_5-6/8-14_ G12V *cis*-spliced peptide and many other peptide products. The latter analysis was carried out by measuring the samples through the Q Exactive Orbitrap mass spectrometer.

This temporal description of the study was detailed in our original article (11). In contrast, Dr. Beer claimed that we first measured the 0 - 4 h *in vitro* digestion kinetics through Q Exactive Orbitrap and that we measured the 20 h *in vitro* digestion samples through Fusion Lumos Orbitrap mass spectrometer a few weeks later. Although no explanation of the grounds for this hypothesis was provided by Dr. Beer (36), we may speculate that Dr. Beer was misled by the dates on the MS RAW files published in the PRIDE repository (PXD015580). In fact, in that PRIDE repository dataset, the Q Exactive Orbitrap measurements have been carried out on May 21^st^, 2019, and the Fusion Lumos Orbitrap measurements have been carried out on June 27^th^ 2019 (**Table S2**).

Unfortunately for Dr. Beer, the published data represented only a tiny portion of all experiments performed. Our conclusions were not only based on one set of experiments but on many different experiments. It should be noted, our original study began in 2014. The first identification of the KRAS_5-6/8-14_ *cis*-spliced G12V peptide was obtained on May 2015, as summarized in the intellectual property-related statement of the inventors of the patent PCT/EP2019/050027. Since then, many *in vitro* digestions and MS measurements have been carried out. Publishing all of them in the PRIDE repository would have been counter to the interest of the readers, the repository and the authors. Therefore, in our original paper (11), we published the most recent MS RAW files in the PRIDE repository dataset PXD015580. We now publish additional Fusion Lumos Orbitrap RAW files of 20 h *in vitro* digestion samples in the PRIDE repository dataset PDX024528 linked to this rebuttal article. These were generated some months prior to the Q Exactive Orbitrap RAW files published in the PRIDE repository dataset PXD015580 associated to our original study (11). The identification of the KRAS_5-6/8-14_ *cis*-spliced G12V peptide through comparison with the cognate synthetic peptide was based on targeted MS through Fusion Lumos Orbitrap (**Figure 5** and **Table S2**). That sample was a 20 h *in vitro* digestion of the synthetic KRAS_2-35_ G12V polypeptide with purified proteasomes. The assay was carried out months before the *in vitro* digestion kinetics measured on the Q Exactive Orbitrap, which Dr. Beer incorrectly thought represented our first assay.

Furthermore, Dr. Beer used his incorrect timeline reconstruction to speculate on why we did not correctly identify the KRAS_5-6/8-14_ G12V *cis*-spliced peptide in the *in vitro* digestion kinetics measured by a Q Exactive Orbitrap whereas we identified the *cis*-spliced peptide in the 20 h *in vitro* digestion samples measured by a Fusion Lumos Orbitrap mass spectrometer. Dr. Beer speculated that the titration of the synthetic KRAS_5-6/8-14_ G12V *cis*-spliced peptide, which were run after the *in vitro* digestion kinetics, “could have resulted in carry-over in the HPLC/MS or in sample cross-contamination during the preparation”. Regarding the latter hypothesis, *i.e.* the kind of sample contaminations proposed by Dr. Beer (36), we showed MS2 spectra that led to the identification of the KRAS_5-6/8-14_ G12V *cis*-spliced peptide produced by proteasomes in the new experiments carried out for this rebuttal article (**Figures 2** and **3**), as well as in former assays (**Figure 5**), which were carried out prior to the assays shown in our original paper [Figure 3 of (11)]. To note, the experiments shown in **Figures 2** and **3** were performed using a different proteasome batch and cell-source, a different reaction buffer and were carried out in a different laboratory than those originally published (11), which strengthened the observation that this spliced peptide is produced by proteasomes.

In the PRIDE repository dataset PDX024528 linked to this rebuttal article, we also provide the MS RAW file of the *in vitro* 0 h digestions of the synthetic KRAS_2-35_ G12V polypeptide with purified proteasomes. This sample was measured through Fusion Lumos Orbitrap mass spectrometer right before the *in vitro* 20 h digestion sample, wherein we identified the KRAS_5-6/8-14_ G12V *cis*-spliced peptide [see Figure 3 in (11)]. In the 0 h *in vitro* digestion sample we did not identify the KRAS_5-6/8-14_ G12V *cis*-spliced peptide.

In our opinion, a sample contamination with the target synthetic peptide, which occurred in various 20 h *in vitro* digestion samples performed and measured in different periods but not occurring in the 0h *in vitro* digestion samples measured in the same periods, would have been very unlikely.

Regarding the former speculation by Dr. Beer, *i.e.* the contamination of the MS runs through a synthetic peptide carry-over in the HPLC/MS, it is worth noting that the synthetic peptide titration mentioned by Dr. Beer was measured on the Q Exactive Orbitrap, whereas the 20 h *in vitro* digestion samples were measured by a Fusion Lumos Orbitrap mass spectrometer. This means that they were measured in two distinct mass spectrometers coupled to two distinct HPLC columns. We are not aware of any example of peptide carry-over occurring between physically separated mass spectrometers.

As a full data disclosure, we also published the list of samples that were measured through both mass spectrometers before and after the MS RAW files provided in our original paper (**Table S3**). No synthetic KRAS_5-6/8-14_ G12V peptide was measured prior to the measurement of the *in vitro* 20 h digestion samples by a Fusion Lumos Orbitrap mass spectrometer and of the 0 - 4 h *in vitro* kinetics samples by a Q Exactive Orbitrap. As mentioned above, we also published some of the MS RAW files of the Fusion Lumos Orbitrap run that preceded the MS runs of the 20 h digestion samples through the Fusion Lumos Orbitrap mass spectrometer originally published in the PRIDE repository dataset PXD015580 (11), as further evidence that Dr. Beer’s speculation was unsubstantiated (see *Data Availability*). For instance, the *in vitro* 0 h digestion of the synthetic KRAS_2-35_ G12V polypeptide with purified proteasomes (J_Liepe_ CB1_no_2_260619_Lumos_rep1.raw; PRIDE PDX024528 dataset; see **Table S3**), which was measured before the *in vitro* 20 h digestion sample “J_Liepe_CB6_yes_2_260619 _Lumos_rep1.raw” (PRIDE PXD015580; see **Table S3**) originally published in (11), did not show evidence of the KRAS_5-6/8-14_ *cis*-spliced G12V peptide.

This is a further evidence that the “peptide carry-over in the HPLC/MS” hypothesis of Dr. Beer was not supported by any experimental proof.

Finally, in his matters arising (36), Dr. Beer repudiated that we could have obtained a better MS2 spectrum of the KRAS_5-6/8-14_ *cis*-spliced G12V peptide in the *in vitro* 20h digestion rather than the 4 h digestions because of a larger degradation of the substrate and production of the target *cis*-spliced peptide in the 20h digestion. Indeed, he claimed that “digestion reached saturation after 3-4 hours” (36). What Dr. Beer noted in **Figure 5C** of our original article was a change in the substrate consumption between 3 h and 4 h (11). As shown there, after 4 h digestion a quarter of the substrate was still present. Dr. Beer might have been misled, and thus estimated, unsupported by the actual data, that the reaction was complete. Various studies have shown that proteasomes change their catalytic dynamics over time. These dynamics changes could be due to the regulation by non-catalytic modifier sites of proteasomes (39–41). Therefore, a modification of substrate degradation trend between two time points should not be considered as a proof of saturation, in contrast to Dr. Beer’s speculation.

In summary, in this section, we illustrate that: (a) Dr. Beer’s speculation of a sample contamination was incorrect and unsubstantiated, and (b) Dr. Beer’s speculation of a different timeline of our study development than that which we originally detailed in (11) was also incorrect and unsubstantiated.

## Discussion

Dr. Beer (36) suggested, implicitly, that the identification of the *cis*-spliced neoepitope was the result of a biased evaluation of the MS results. In our opinion, his deductions were based on a misinterpretation of our raw datasets, and no reliable further experiments supporting his hypothesis.

In our rebuttal article, we show in detail how and why Dr. Beer’s conclusions are wrong. In fact, the *cis*-spliced peptide was produced by proteasomes and was not the result of the kind of contaminations proposed by Dr. Beer (36). Further studies are needed to determine the presentation of the KRAS_5-6/8-14_ G12V *cis*-spliced peptide at the cell surface of cancer cells, and its suitability as target for anti-cancer immunotherapy.

Furthermore, we provided various lines of evidence that our original narrative (11) describing the *in silico/in vitro* pipeline and the identification of the KRAS_5-6/8-14_ G12V *cis*-spliced peptide were correct, in contrast to Dr. Beer’s alternative reconstruction of our study.

Nonetheless, this rebuttal article motivated us to explore alternative approaches for the identification of target spliced neoepitope candidates. Despite the success in identifying the KRAS_5-6/8-14_ G12V *cis*-spliced peptide using standard MS (**Figures 2** and **3**), we still considered that a targeted MS strategy - informed by an *in silico* pipeline such as that which we previously described (11) – may lead to a more robust identification of the target neoepitope candidates produced by proteasomes.

## Author Contributions

MM and JL developed the project, performed and supervised the data analysis and data generation, and wrote the manuscript. GR-H performed experiments. HU critically validated the data analysis, and performed and supervised the MS measurements. JN edited the manuscript and critically evaluated the results. All authors contributed to the article and approved the submitted version.

## Funding

The study was in part supported by: (i) MPI-BPC collaboration agreement 2020, Cancer Research UK [C67500; A29686] and National Institute for Health Research (NIHR) Biomedical Research Centre based at Guy’s and St Thomas’ NHS Foundation Trust and King’s College London and/or the NIHR Clinical Research Facility to MM; (ii) ERC-StG 945528 IMAP to JL.

## Conflict of Interest

MM and JL are co-inventors of the spliced neoepitope and specific TCRs protected by the patent PCT/EP2019/050027 and US16/958,604.

The remaining authors declare that the research was conducted in the absence of any commercial or financial relationships that could be construed as a potential conflict of interest.
