# Supplementary material for: Response: Commentary: An In Silico–In Vitro Pipeline Identifying an HLA-A*02:01+ KRAS G12V+ Spliced Epitope Candidate for a Broad Tumor-Immune Response in Cancer Patients
Source: Front Immunol. 2021 Jul 13;12:679836. doi: 10.3389/fimmu.2021.679836 (PMC8315000; doi:10.3389/fimmu.2021.679836)
Supplement: Supplementary file 1 [file DataSheet_1.pdf]

Confirmed identification of an HLA-A*02:01+ KRAS G12V+ *cis*-spliced peptide produced by proteasomes

Correspondence to: michele.mishto@kcl.ac.uk, jliepe@mpibpc.mpg.de.

Table S1	MS scans reanalyzed in Beer's matters arising
Table S2	Orbitraps' MS2 spectra assigned to the $KRAS_{5-6/8-14}$ G12V *cis*-spliced peptide and the $KRAS_{5-13}$ G12V non-spliced peptide.
Table S3	List of samples that were measured through four different mass spectrometers before and after the MS RAW files provided in our original paper [1] and in the present study
Figure S1	$KRAS_{5-6/8-14}$ G12V *cis*-spliced and $KRAS_{5-13}$ G12V non-spliced peptides' MS ion chromatograms of *in vitro* digestion kinetics measured through Q Exactive HF Orbitrap
Figure S2	$KRAS_{5-6/8-14}$ G12V *cis*-spliced and $KRAS_{5-13}$ G12V non-spliced peptides' MS ion chromatograms of *in vitro* digestion kinetics measured through Q Exactive Orbitrap

Table S1. MS scans reanalyzed in Beer's matters arising. Here, the details of the three MS scans (11558, 11683, 11389) - discussed in Beer's matters arising [2], and generated for our original study [1] – are reported. The file is accessible in the journal's website.

Table S2. Orbitraps' MS2 spectra assigned to the KRAS$_{5\text{-}6/8\text{-}14}$ G12V *cis*-spliced peptide and the KRAS$_{5\text{-}13}$ G12V non-spliced peptide. Information about the MS2 spectra generated by Exploris 480 Orbitrap, Q Exactive HF and Fusion Lumos Orbitraps and assigned to the KRAS$_{5\text{-}6/8\text{-}14}$ G12V *cis*-spliced peptide and the KRAS$_{5\text{-}13}$ G12V non-spliced peptide by our pipeline. The file is accessible in the journal's website.

Table S3. List of samples that were measured through four different mass spectrometers before and after the MS RAW files provided in our original paper [1]. List of samples that were measured through: (i) the Q Exactive Orbitrap and Fusion Lumos Orbitrap before and after the MS Raw files published in the PRIDE repository (PXD015580) associated to our original study [1]; (ii) the Exploris 480 and Q Exactive HF Orbitraps before and after the MS Raw files published in the PRIDE repository (PDX024528) associated to our rebuttal article. Only samples related to our rebuttal article have been listed. To note, we have never measured the synthetic KRAS$_{5\text{-}6/8\text{-}14}$ peptide prior *in vitro* digestions. The file is accessible in the journal's website.

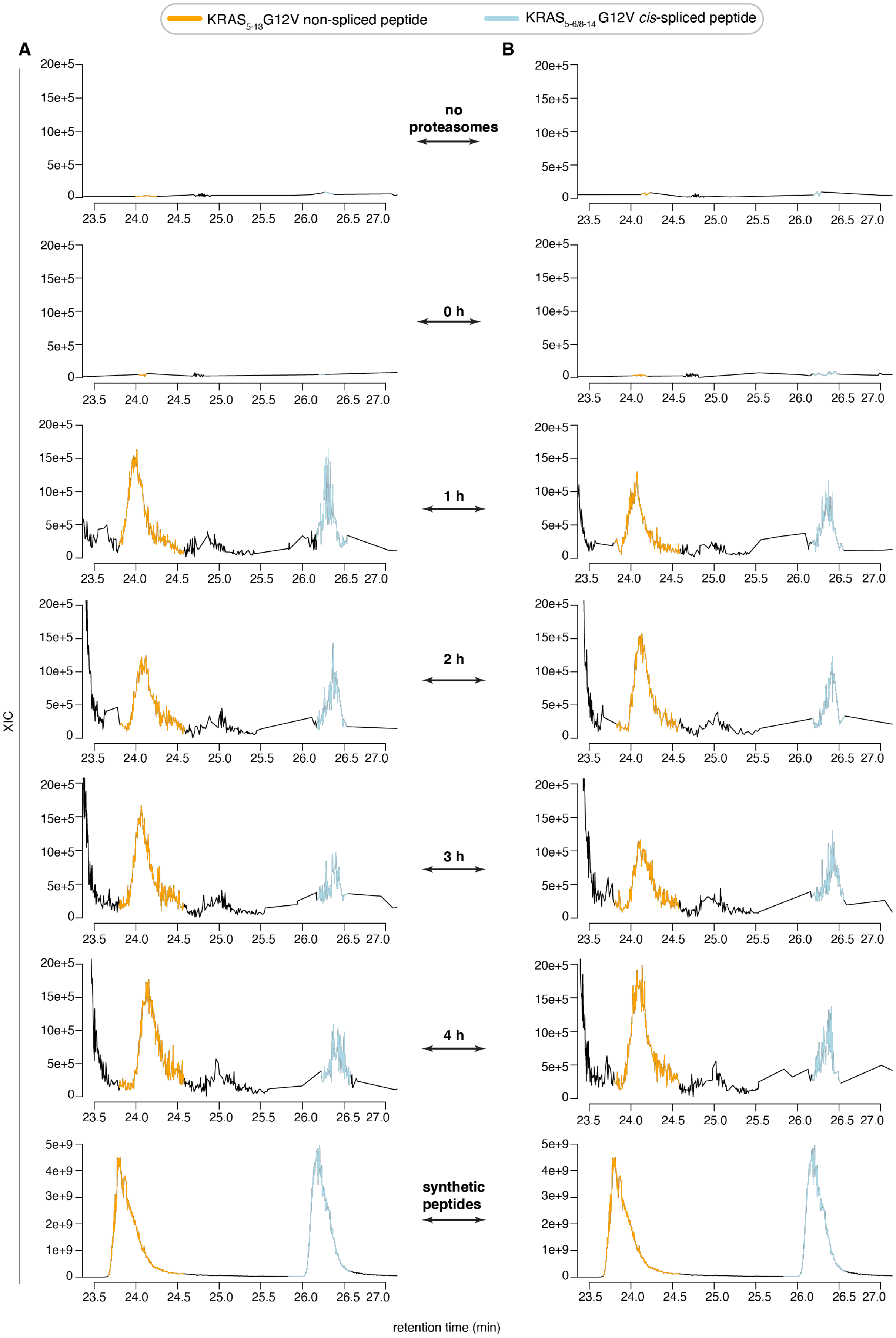

KRAS5-13G12V non-spliced peptide
KRAS5-6/8-14G12V cis-spliced peptide
A
B
no proteasomes
0 h
1 h
2 h
3 h
4 h
synthetic peptides
XIC
retention time (min)

Figure S1. KRAS$_{5-6/8-14}$ G12V *cis*-spliced and KRAS$_{5-13}$ G12V non-spliced peptides' MS ion chromatograms of *in vitro* digestion kinetics measured through Q Exactive HF Orbitrap. (**A,B**) MS extracted ion chromatograms (for the m/z = 421.275 – 421.875) of *in vitro* digestion kinetics (0-4 h) of the synthetic polypeptide KRAS$_{2-35}$ G12V with purified proteasomes named as "MF" (**A**) and "MG" (**B**) series. The peak corresponding to the KRAS$_{5-6/8-14}$ G12V *cis*-spliced and KRAS$_{5-13}$ G12V non-spliced peptides are color labelled. For comparison, the synthetic peptide counterparts are plotted and indicate matching RTs. Samples were measured through Q Exactive HF Orbitrap. Synthetic peptides have been measured after the *in vitro* digestion kinetics (**Table S3**).

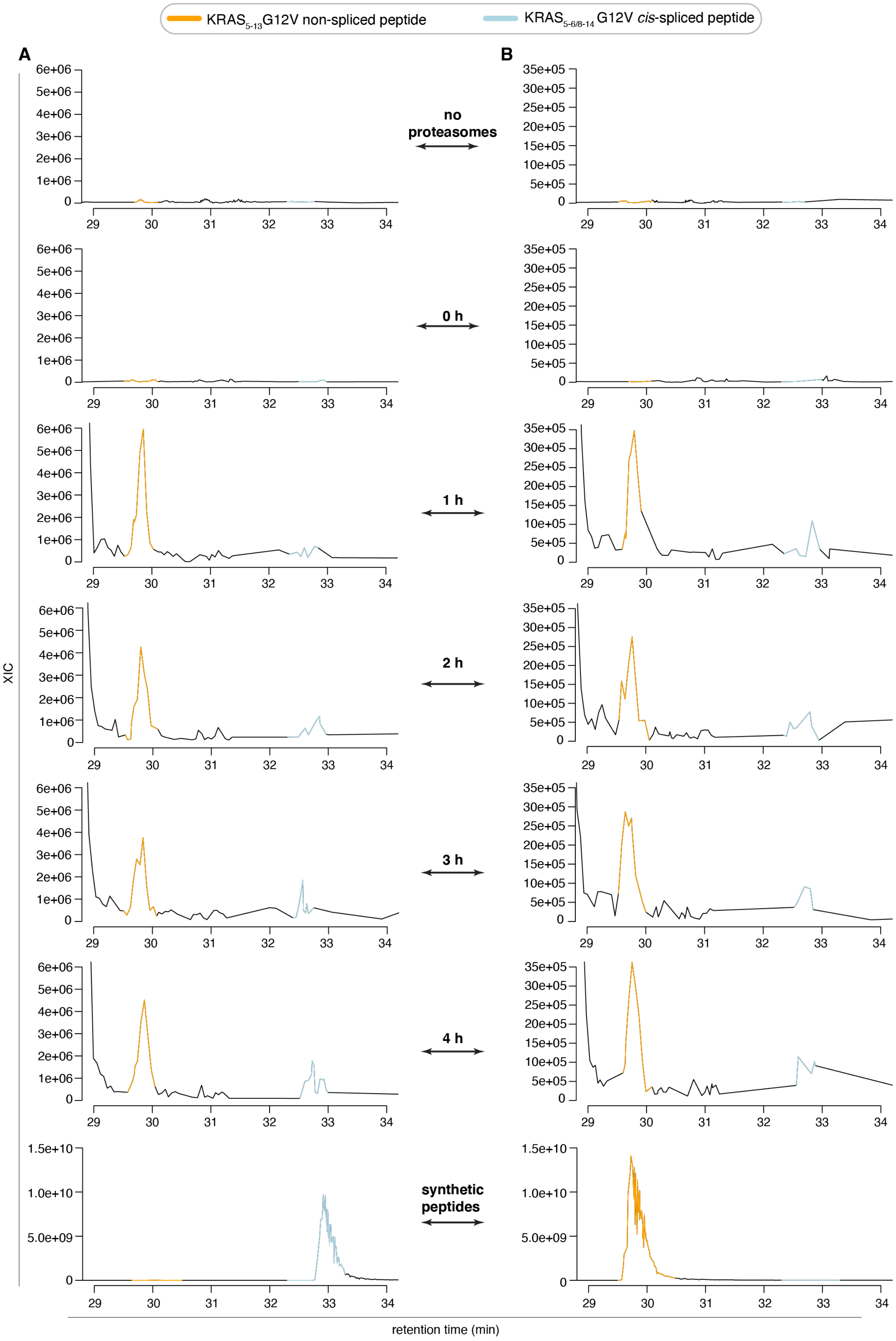

KRAS$_{5-13}$G12V non-spliced peptide
KRAS$_{5-6/8-14}$G12V *cis*-spliced peptide
A
B
no proteasomes
0 h
1 h
2 h
3 h
4 h
synthetic peptides
XIC
retention time (min)

Figure S2. $KRAS_{5-6/8-14}$ G12V *cis*-spliced and $KRAS_{5-13}$ G12V non-spliced peptides' MS ion chromatograms of *in vitro* digestion kinetics measured through Q Exactive Orbitrap. (**A,B**) MS extracted ion chromatograms (for the m/z = 421.275 – 421.875) of *in vitro* digestion kinetics (0-4 h) of the synthetic polypeptide $KRAS_{2-35}$ G12V with purified proteasomes named as "MF" (**A**) and "MG" (**B**) series. The peak corresponding to the $KRAS_{5-6/8-14}$ G12V *cis*-spliced and $KRAS_{5-13}$ G12V non-spliced peptides are color labelled. For comparison, the synthetic peptide counterparts (measured singularly as in opposite to **Fig. 4**) are plotted and indicate matching RTs. Samples were measured through Q Exactive Orbitrap and the same method as in the original study [1]. Synthetic peptides have been measured after the *in vitro* digestion kinetics (**Table S3**).

Reference

[1] M. Mishto, A. Mansurkhodzhaev, G. Ying, A. Bitra, R.A. Cordfunke, S. Henze, D. Paul, J. Sidney, H. Urlaub, J. Neefjes, A. Sette, D.M. Zajonc, and J. Liepe, An in silico-in vitro Pipeline Identifying an HLA-A(*)02:01(+) KRAS G12V(+) Spliced Epitope Candidate for a Broad Tumor-Immune Response in Cancer Patients. Front Immunol 10 (2019) 2572.

[2] I. Beer, Commentary: An in silico-in vitro Pipeline Identifying an HLA-A*02:01+ KRAS G12V+ Spliced Epitope Candidate for a Broad Tumor-Immune Response in Cancer Patients. Front Immunol (2021).
